# Predicting Rice Heading Date Using an Integrated Approach Combining a Machine Learning Method and a Crop Growth Model

**DOI:** 10.3389/fgene.2020.599510

**Published:** 2020-12-18

**Authors:** Tai-Shen Chen, Toru Aoike, Masanori Yamasaki, Hiromi Kajiya-Kanegae, Hiroyoshi Iwata

**Affiliations:** ^1^Graduate School of Agricultural and Life Sciences, The University of Tokyo, Bunkyo, Japan; ^2^Food Resources Education and Research Center, Graduate School of Agricultural Science, Kobe University, Kasai, Hyogo, Japan

**Keywords:** crop growth model, bayesian inference, differential evolution adaptive metropolis, machine learning, Markov chain Monte-Carlo

## Abstract

Accurate prediction of heading date under various environmental conditions is expected to facilitate the decision-making process in cultivation management and the breeding process of new cultivars adaptable to the environment. Days to heading (DTH) is a complex trait known to be controlled by multiple genes and genotype-by-environment interactions. Crop growth models (CGMs) have been widely used to predict the phenological development of a plant in an environment; however, they usually require substantial experimental data to calibrate the parameters of the model. The parameters are mostly genotype-specific and are thus usually estimated separately for each cultivar. We propose an integrated approach that links genotype marker data with the developmental genotype-specific parameters of CGMs with a machine learning model, and allows heading date prediction of a new genotype in a new environment. To estimate the parameters, we implemented a Bayesian approach with the advanced Markov chain Monte-Carlo algorithm called the differential evolution adaptive metropolis and conducted the estimation using a large amount of data on heading date and environmental variables. The data comprised sowing and heading dates of 112 cultivars/lines tested at 7 locations for 14 years and the corresponding environmental variables (day length and daily temperature). We compared the predictive accuracy of DTH between the proposed approach, a CGM, and a single machine learning model. The results showed that the extreme learning machine (one of the implemented machine learning models) was superior to the CGM for the prediction of a tested genotype in a tested location. The proposed approach outperformed the machine learning method in the prediction of an untested genotype in an untested location. We also evaluated the potential of the proposed approach in the prediction of the distribution of DTH in 103 F_2_ segregation populations derived from crosses between a common parent, Koshihikari, and 103 cultivars/lines. The results showed a high correlation coefficient (ca. 0.8) of the 10, 50, and 90th percentiles of the observed and predicted distribution of DTH. In this study, the integration of a machine learning model and a CGM was better able to predict the heading date of a new rice cultivar in an untested potential environment.

## Introduction

Heading date is a critical trait for the adoption of a rice cultivar to target cultivation area and cropping season (Yano et al., 1997). Improvement in the understanding and modeling of rice phenology could benefit production and breeding. However, it has been challenging to model a complex trait such as heading date, which is usually influenced by genotype, environment, and their interaction. In the past, when available data were limited, simple models such as those based on growing degree days have been used widely, but these have good predictability for the specific genotype in environments with few variabilities. With more available data and knowledge and the improvement in computing power, more complex models such as crop growth models (CGMs) have been developed to simulate the performance of genotypes in a wide range of environments, mainly variable temperature and photoperiod. A CGM is implemented as a process-based mathematical set of equations describing the growth process of a crop plant, and enables the prediction of growth and production under environmental, management, and physiological input variables. The physiological parameters in the CGM equations account for the among-genotype differences and are usually regarded as environment-independent genotypic characteristics (Yin et al., 2000). This allows the predictions to be unrestricted to environments where the model parameters are calibrated/estimated (Yin et al., 2003).

As genetic marker information becomes available, the genetic control of the response to environments can be revealed via the dissection of the variation in the CGM parameters into the effects of discrete genetic loci—quantitative trait loci (QTLs). The relevant studies include the research on flowering time in barely (Yin et al., 2005), rice (Nakagawa et al., 2005), *Brassica oleracea* (Uptmoor et al., 2008), and wheat (Bogard et al., 2014). These studies suggest the possibility of predicting the performance of a given genotype in an untested environment by plugging in the parameters that are predicted for the genotype based on the estimated QTL effects into a CGM. As an example, Bogard et al. (2014) predicted days to heading (DTH) of wheat based on the estimated QTL effects and found that the root mean square error (RMSE) between the observed and predicted values was 6.3 days. The approach of integrating a gene-based or QTL-based model with a CGM has been advocated by several studies (White and Hoogenboom, 1996; Chapman et al., 2002a,b; Letort et al., 2008). However, further refinement is required for linking the CGM parameters with genotypes of markers or genes.

For the integrated approach, we must first estimate the parameters of the CGM using the phenotypic and environmental data collected in field experiments. Owing to several reasons, such as the lack of sufficient input data for estimating many parameters, difficulties in defining the criteria for validating the predicted accuracy of a CGM, and the diverse structure of input data, the estimation of CGM parameters remains a rather open field (Seidel et al., 2018). The estimation methods can be classified as frequentist or Bayesian. The frequentist approach assumes that the parameter is a fixed effect and does not include the prior information of the parameter in the model. The Bayesian approach assumes that the parameter is a random variable and the prior information is built into the model. A comprehensive introduction of this topic can be found in Makowski et al. (2006). Although the better choice among Bayesian and frequentist approaches is not clear, the Bayesian approach could provide further information regarding the parameters, such as the uncertainty of the estimates, when the main interest is in interpreting the biological meaning of estimated parameter values instead of optimizing the predicted accuracy of the CGM. In several studies (Iizumi et al., 2009; Jones et al., 2011) a Bayesian approach with the Markov chain Monte Carlo (MCMC) technique has been applied for estimating CGM parameters. The commonly used MCMC method, such as the Metropolis–Hastings algorithm, however, has slow convergence in practice. Dumont et al. (2014) and Iizumi et al. (2014) suggested the use of an advanced MCMC technique, such as the differential evolution adaptive metropolis (DREAM) algorithm, which can automatically tune the scale and orientation of the proposed distribution during the search and overcome the problems of heavy-tailed and multimodal posteriors.

Another consideration is how an integrated framework connecting the CGM to markers or genes can be built for predicting complex traits. A straightforward approach is the two-step approach that first computes the estimates of the CGM parameters and then uses the statistical models developed for QTL analysis or genomic prediction (Meuwissen et al., 2001) to predict the CGM parameters. A unified predictive system has also been proposed by Technow et al. (2015) and Onogi et al. (2016) Their framework applied different Bayesian approaches, but both based their system on a single hierarchical model instead of the two-stage approach to predict complex traits such as yield in maize and heading in rice, respectively. Although the integration of genomic prediction with CGM has shown good potential in previous studies, another modeling paradigm, such as machine learning, could also have great potential as a candidate method for modeling the non-linear, complicated interaction between the gene and the environment.

Unlike statistical models that focus more on the extraction of information on the underlying mechanism producing the data, the machine learning method is concerned with the accuracy of prediction (Breiman, 2001b). As a result of the big data era, machine learning has shown unprecedented predictive power against traditional statistical models. However, there were very few studies applying the machine learning method in predicting crop growth, which could stem from the lack of appropriate data and unfamiliarity with this method in the relevant community. In this study, we collected the heading data of 112 rice cultivars/lines tested in multiple locations from 2004 to 2017. This large amount of heading data combined with environmental data and genetic marker data allowed us to train a robust machine learning model and to compare its predictability with that of other methods. We also collected the heading of 103 F_2_ segregating populations created from the crosses of cultivars/lines, which were selected from the 112 cultivars/lines. This F_2_ population data helps validate the model performance in predicting DTH of a simulated genotype in a new environment. In addition to training a single machine learning model, building an integrated framework combining a CGM and a machine learning model to predict the complex trait could also be a promising method that has not been attempted earlier.

In this study, we explored the potential use of the machine learning method and proposed an integrated approach that could be superior in an interpolation scenario. We implemented a Bayesian method for the estimation of CGM parameters. Although many powerful machine learning methods have been proposed, there is no single best method that can outperform others on all fronts, such as the so-called “no free lunch” theorem. In this study, we evaluated three representative methods: two decision tree-based approaches [random forest (RF) and eXtreme gradient boosting (XGB)], and a neural network-based approach [extreme learning machine (ELM)]. We compared the predictive performance of different modeling methods, including a CGM [developmental rate (DVR) model], three machine learning methods, and the proposed integrated framework, which combines machine learning and CGM using a two-stage approach to predict the DTH in rice. The comparison was performed under three cross-validation schemes. We also examined the ability of the proposed integrated framework in predicting the distribution of DTH in 103 F_2_ segregation populations and demonstrated the superiority of the proposed approach in predicting the heading date of a new genotype in a new environment.

## Materials and Methods

### Rice Heading Data

Two datasets of experiments evaluating DTH in rice cultivars/lines were analyzed in this study. The first was the dataset of 112 cultivars/lines, and the other was the dataset of F_2_ segregation populations derived from crosses between a Japanese leading cultivar as a common parent, Koshihikari, and 103 cultivars/lines. The 112 cultivars/lines dataset comprised 7,098 observations of sowing, transplanting, and heading dates of the 112 cultivars/lines evaluated in eight locations in Japan from 2004 to 2017 (64 combinations of locations and years in total, Supplementary Table S1). The 112 cultivars/lines were chosen from those developed in different regions of Japan (Supplementary Table S2). The experiments were conducted in one location (Tsukubamirai) in the middle of Japan in the first 2 years, and then gradually expanded to other locations distributed from the north to the south of Japan in the following years. All 112 cultivars/lines were sown and transplanted at the same time in a single experiment at each location, and more than one experiment (sowing and transplanting on different dates) was conducted at some locations. We defined the heading date as the date when more than 50% of individuals reached the heading stage. The number of plants evaluated for each cultivar/line was different among the experiments and ranged from 7 to 30. DTH was calculated as the difference between the heading date and sowing date. In 70 of 7,168 cases, cultivars/lines did not reach the heading stage before the end of the experiment. Thus, 70 cases were removed from the analysis. The dataset of the F_2_ segregation population was created by crossing Koshihikari and 103 of the 112 cultivars/lines. In 2007 and 2008, we evaluated 73 and 30 F_2_ populations, respectively, in Kasai, Hyogo. Each population was evaluated using 96 F_2_ plants (genotypes). The distribution of DTH in each segregation population was obtained by recording the heading date of each plant individually.

### Meteorological Data

Temperature and photoperiod (day length) are the two most influential meteorological factors affecting the phenological development (e.g., flowering) of rice. We downloaded the daily average temperature data from the Agro-Meteorological Grid Square Data, National Institute for Agro-Environmental Sciences, National Agriculture and Food Research Organization, Japan. We computed the theoretical day length based on the latitude and longitude of each location according to the CBM model (Forsythe et al., 1995).

### Genotype Marker Data

We used two sets of genotypic marker data from 112 cultivars/lines in this study. The first was the genotype data of 14 SNPs in five heading date-related genes, *Hd1* (Yano et al., 2000), *Ghd7* (Xue et al., 2008), *Hd6* (Takahashi et al., 2001), *Hd16* (Hori et al., 2013), and *Hd17* (Matsubara et al., 2012). The other was the genotype data of 1,594 markers, which included the 14 heading date-related SNPs and other SNPs and Simple-sequence repeats (SSRs) markers. We generated 1,000 simulated genotypes of the 14 heading date-related SNPs as simulated progeny from each F_2_ population. The simulation was performed based on the linkage map positions of the SNPs and genotype marker data of parents of an F_2_ population.

### Methods for Predicting Rice Heading

We compared three methods in the prediction of the heading date of rice. CGM, a machine learning method, and the proposed integrated models. The three methods are described in Table 1 with the type of input data and the type of cross-validation schemes, which are explained in section “Cross-Validation.”

**TABLE 1:** Prediction methods used in the study.

**Method^a^**			**Input^b^**		**Cross-validation^c^**	
**Type**	**Name**	**Description**	**E**	**G**	**LOGO**	**LOGLO**
CGM	DVR	DVR model with Bayesian DREAM MCMC algorithm	✓			
Machine learning	ELM	Extreme learning machine	✓	✓		
	XGB	Gradient boosting	✓	✓	✓	✓
	RF	Random forest	✓	✓		
Integrated model	CGM-ELM	DVR-Bay -> ELM	✓	✓	✓	✓
	CGM-XGB	DVR-Bay -> GB	✓	✓	✓	✓
	CGM-RF	DVR-Bay -> RF	✓	✓	✓	✓

*^*a*^Methods used for predicting days to heading in rice.^*b*^Input data used for prediction: E indicates environmental data, including daily mean temperature and daily photoperiod; G indicates the genotype marker data.^*c*^LOGO represents leave-one-genotype-out cross-validation. LOGLO is a leave-one-combination-of-genotype-and-location-out cross-validation.*

### DVR Model

A CGM named the DVR model was modified from a three-stage beta model (Yin et al., 1997), as proposed by Nakagawa et al. (2005). The model assumes that the pre-flowering development of a rice plant is divided into three subphases: (1) the juvenile phase, when the plant is insensitive to the flowering stimulus; (2) the “photoperiod sensitive phase,” when the plant starts to respond to the photoperiodic flowering stimulus; and (3) the “post- photoperiod sensitive phase,” the period after the completion of the photoperiod sensitive phase. The progress of developmental stages (DVS) from seedling emergence (DVS0), flowering (DVS1) to maturation (DVS2) is quantified as 0, 1, and 2, respectively, and is calculated by integrating the growth rate of the *i*-th day *DVR*_*i*_ as:

$$D\,V\,S_{d}=\sum_{i=0}^{d}D\,V\,R_{i}$$

where *d* is the number of days since seedling emergence. DVR is modeled as the multiplicative function of a temperature response function and a photoperiod response function, and is defined as follows:

$$D\,V\,R_{i}=\left\{ \begin{array}{c} \frac{f\,\left(T_{d}\right)}{G}\;i\,f\,D\,V\,S_{d}\,<D\,V\,S_{1}\,o\,r\,D\,V\,S_{d}>\,D\,V\,S_{2}\\ f\,\left(T_{d}\right)\,g\,\left(P_{d}\right)/G\;i\,f\,D\,V\,S_{1}<D\,V\,S_{d}<D\,V\,S_{2} \end{array}\right.$$

where *T*_*d*_ and *P*_*d*_ are the daily mean temperature (°C) and the photoperiod (h) of the *d*-th day, respectively, *f* and *g* denote the temperature response function and photoperiod function, respectively, and *G* (*G* > 0) denotes the earliness of flowering under the optimal condition. *DVS*_*1*_ and *DVS*_*2*_ represent the ends of the juvenile and photosensitive phases, respectively. The functions *f* and *g* are given by

$$f\,\left(T_{d}\right)=$$

$$\{\begin{array}{c} {\left[\left(\frac{T_{d}-T_{b}}{T_{o}-T_{b}}\right)\,{\left(\frac{T_{c}-T_{d}}{T_{c}-T_{o}}\right)}^{\left(T_{c}-T_{o}\right)/\left(T_{o}-T_{b}\right)}\right]}^{\alpha }\,i\,f\,T_{b}\leq T_{d}\leq T_{c},\\ 0\;o\,t\,h\,e\,r\,w\,i\,s\,e \end{array}$$

$$g\,\left(P_{d}\right)=\left\{ \begin{array}{c} {\left[\left(\frac{P_{d}-P_{b}}{P_{o}-P_{b}}\right)\,{\left(\frac{P_{c}-P_{d}}{P_{c}-P_{o}}\right)}^{\frac{P_{c}-P_{o}}{P_{o}-P_{b}}}\right]}^{\beta }\,i\,f\,P_{o}\leq P_{d},\\ 1\;o\,t\,h\,e\,r\,w\,i\,s\,e \end{array}\right.$$

where, *T*_*b*_, *T*_*c*_, and *T*_*o*_ are the base, ceiling, and optimum temperatures (in the unit of degree Celsius), respectively, and *P*_*b*_, *P*_*c*_, and *P*_*o*_ represent the base, ceiling, and optimum photoperiods (in the unit of hours), respectively. The values of *T*_*b*_, *T*_*o*_, *T*_*c*_, *P*_*b*_, *P*_*o*_, and *P*_*c*_, are fixed at 8, 30, 42, 0, 10, and 24 according to Nakagawa et al. (2005). The parameter α (α > 0) is the temperature-sensitivity coefficient, whereas β (β > 0) is the photoperiod sensitivity coefficient. To minimize the number of parameters, *DVS*_*1*_ and *DVS*_*2*_ are defined as

$$D\,V\,S_{1}=0.145+0.005\,G$$

$$D\,V\,S_{2}=0.345+0.005\,G$$

according to Nakagawa et al. (2005). Parameters α, β, and *G* remain in the DVR model and are assumed to be able to quantify genetic differences in phenological responses to environmental factors.

### Parameter Estimation

We used the advanced MCMC algorithm to estimate the posterior distribution of the parameters (α, β, and *G*). The details of the implemented DREAM algorithm are provided in Supplementary Material. The DREAM algorithm runs multiple chains instead of a single chain. The number of chains should be larger than twice the number of parameters (three in the DVR model) and was set to 10. The number of iterations, the number of samples discarded during burn-in, and the number of selected samples were set as 50,000, 10,000, and 10,000, respectively. For the parameters in the DVR model, the normal priors and ranges of the parameters assumed in the study are summarized in Table 2. We developed a program in the language Julia to implement the DREAM algorithm for parameter estimation of the DVR model. The source code is available from the authors upon request.

**TABLE 2:** DVR model parameters and their prior information.

**Parameter**	**Definition**	**Prior N (μ, σ)**	Range*	**Unit**
alpha	Sensitivity of temperature	N (3, 1)	0–20	−
beta	Sensitivity of photoperiod	N (4, 1)	0–25	−
G	Earliness of flowering under optimal photoperiod and temperature	N (35, 2)	30–120	Day

* A proposal that fell out of the range was discarded during Markov chain Monte-Carlo sampling.

### Machine Learning Methods

We implemented RF, XGB, and ELM to predict the heading date of rice. The same training data, with the environmental data and genotypic data as inputs and DTH as outputs, were prepared for the three machine learning methods. The environmental data of each observation consisted of daily temperature from the date of sowing to 199 days later and the daily photoperiod at the sowing day, and 100 and 200 days after sowing. As the theoretical photoperiod has a bell-shaped curve determined only by latitude and longitude, the photoperiod of three representative days was used to avoid multicollinearity in the input variables. As described in section “Genotype Marker Data,” we used two types of genotype marker data. The data were converted to dummy variables and combined with environmental data as input.

RF is an ensemble learning method that combines de-correlated trees and aggregates their predictions by averaging (Breiman, 2001a). It has been successful as a general-purpose classification and regression method and is involved in various practical problems (Biau and Scornet, 2016). We implemented RF using the R package “randomForest” (Liaw and Wiener, 2002) with hyperparameters set as the default values, except for the following parameters: the number of trees *ntree=500* and the number of variables randomly sampled *mtry* = *p*/3, where p is the number of columns in the input matrix.

The gradient tree boosting proposed by Friedman (2002) is an effective and popular machine learning method. Chen and Guestrin (2016) and Sagi and Rokach (2018) implemented a scalable end-to-end tree boosting system, called XGB, which includes innovations such as a novel tree learning algorithm and a theoretically justified weighted quantile sketch procedure. XGB has won competitions for machine learning on Kaggle (Ziêba et al., 2016) and has been proven to be a versatile and effective tool in regression and classification problems. We implemented XGB using the R package “XGBoost” (Chen and He, 2015) with hyperparameters set as their default values except the following parameters: the maximum depth of a tree = 6, learning rate = 0.1, and the number of iterations = 200.

An ELM is a single hidden layer neural network that randomly assigns the hidden node learning parameters and analytically determines the network output weights by solving the linear square system using the least squares method (Huang et al., 2006). ELM can save time in the training process compared to a feedforward neural network that adjusts weights through a back-propagation method. We implemented ELM using the R package “elmNNRcpp” and set the hyperparameter for the number of hidden nodes as 100 based on the result of a grid search for 25, 50, 100, 200, and 400 nodes.

### Integrated Approach

**FIGURE 1:**
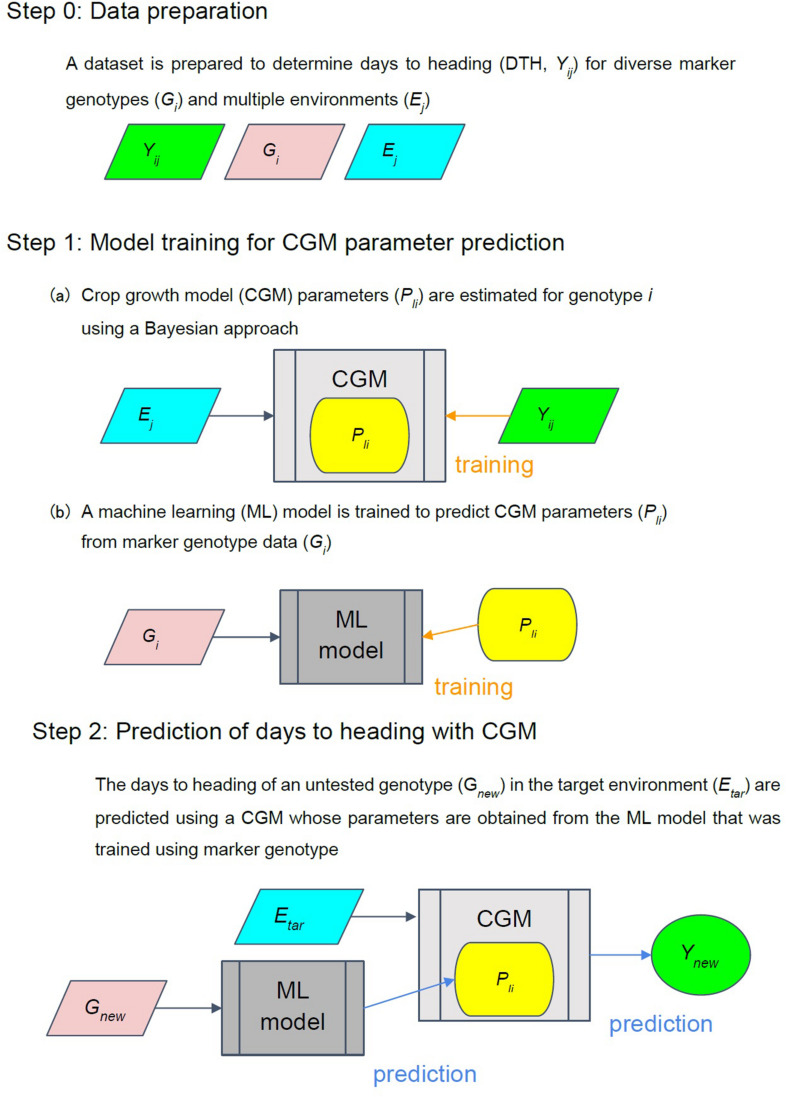
Integrated approach concept. *E*_*j*_ is the environmental data that comprises the daily average temperature and daily photoperiod in the *j*-th environment (from the date of seeding to the date of heading + 70 days). *Y*_*ij*_ is the observed phenotypic trait (heading date) for the *i*-th genotype in *j*-th environment (*j* = 1,2,…,*n*). *P*_*li*_ is the *l*-th CGM parameters for the *i*-th cultivars/line. *G*_*i*_ is a vector of marker genotypes of the *i*-th cultivar/line.

The proposed integrated approach aimed to link the genotypic effect on phenological growth using the concept shown in Figure 1. Data with a large variation in phenological growth among diverse genotypes tested in multiple environments are essential for the success of the proposed approach. The approach is basically a two-step model (Nakagawa et al., 2005; Bogard et al., 2014) that first links the gene effect to the parameters in the CGM through a machine learning method, and then predicts the heading date of a genotype in a target environment through the CGM. In step 1a, we estimated the model parameters (α, β, and *G*) for each genotype using the Bayesian method. The posterior distributions of the parameters are obtained via the Bayesian methods. The mean values of the posterior distributions were chosen as the estimates of the parameters in the Bayesian method. To link the effect of markers to the model parameters, we used 112 cultivars/lines for 14 heading-related markers or 1,594 markers as the input of a machine learning model for predicting the parameter values. Estimates of the parameters were used as the output for training a machine learning model (step 1b). Then, we connected the genetic effect on the parameters to the crop model in step 2 and predicted the heading date of a given marker genotype under the target environment.

### Cross-Validation

Three types of cross-validation (CV) were performed to compare the prediction ability of the different methods. The first is a fivefold CV that was applied to compare the performance of the CGM with the machine learning methods when information on all genotypes and locations are available. This is a scheme used to validate the accuracy of prediction for tested genotypes under tested locations. In a breeding program, we usually do not test the full set of genotypes across all the environments. The prediction under this scheme therefore allows breeders to predict the DTH of “untested combinations” of tested genotypes under the tested locations. Based on the prediction, breeders can evaluate the potential adaptation of a tested genotype to a tested target location.

The second is the leave-one-genotype-out (LOGO) CV. In this scheme, from among the 112 genotypes, one genotype is removed from the data and the model is trained to predict the DTH for the removed genotype. The process is repeated until each genotype has been removed and predicted once. The predictions under this scheme allow breeders to predict the DTH of new lines (or even simulated marker genotypes) under the tested locations. Based on the prediction, breeders can evaluate the potential adaptation of an untested genotype (e.g., lines under development) to a tested target location based on the marker genotype of the untested genotype. The LOGO CV was only applied to machine learning methods, and the integrated approach as the crop model requires the data of the target genotype to estimate the model parameters.

The third is the leave-one-combination-of-genotype-and-location-out (LOGLO) CV. In this scheme, one of the eight locations and one of the 112 cultivars/lines were removed from the data, and the DTH of the removed genotype in the removed location is predicted using the prediction model derived from the data comprising 111 genotypes in 7 locations. This is a scheme to validate the accuracy of prediction of untested genotypes under untested locations. The prediction under this scheme allows us to predict the DTH of new breeding lines (or simulated marker genotypes, as demonstrated in this study) to an untested target environment (e.g., expected environmental conditions in the future) based on marker genotypes of the untested genotype and environmental data of the untested environment.

In each CV scheme, the predicted DTH was obtained for each genotype in each environment (combination of location, year, and treatment of different sowing dates). We then compared the prediction ability among the modeling methods based on the RMSE between the predicted and observed DTH.

### Prediction of DTH in the F_2_ Segregation Populations

The selection of a good parental combination that has a high probability of generating offspring with desired characteristics is important in breeding (Iwata et al., 2013). The better prediction of DTH in the F_2_ segregation population can help the breeder to choose the best parental combination to generate progeny with the desired DTH prior to crossing. This can greatly reduce the cost and increase the efficiency of breeding. To demonstrate the potential of the integrated approach, we implemented the integrated model CGM-XGB trained by parents’ data (the same data of 112 cultivars) and predicted the DTH in the derived F_2_ segregation populations (created from the crossing of selected parental combinations of 112 cultivars) grown under an untested location, Kasai. In Kasai, 103 F_2_ segregation populations derived from a common parent, Koshihikari, and 103 cultivars/lines were planted in 2008 and 2009 with 73 and 30 populations, respectively. We evaluated 96 F_2_ individuals of each segregation population and measured their heading date to determine the distribution of DTH in the population. To predict the distribution of DTH in the segregation populations, we simulated the genotype marker data of F_2_ segregation populations, and then predicted the heading date of simulated genotypes at an untested location with environmental data. The genotype marker data of progeny in an F_2_ segregation population can be simulated from the genotype marker data of their parents and the estimated recombination rates between markers. In this study, we simulated 1,000 progeny for 14 markers of heading date-related genes, and applied the genotype data to the CGM-XGB model constructed based on the data of 112 cultivars/lines to predict the segregation distribution of DTH in the F_2_ population. We considered the range of DTH of the F_2_ segregation population for the selection of progeny with a reasonable value. We therefore compared the 10, 50, and 90th quantiles of the predicted and observed DTH.

## Results

### Estimation of the DVR Model Parameter

We implemented a Bayesian method for estimating the CGM parameters in this study. The Bayesian method provided us with an approximated posterior distribution that was more informative than the point estimation obtained from the frequentist method. Table 3 shows the average of the posterior mean, median, and mode of the CGM parameters (α,β, and *G*) among the cultivars/lines from each origin. The average of the median and mean values of the cultivars/lines from the same origin are similar; however, the average of the mode occasionally deviates from the average of the mean. This tendency is mainly because of the multimodal posteriors induced by the correlation between the CGM parameters. Therefore, the mean of the approximated posterior distribution could be more appropriate to describe the phenological features of a genotype. The genotypes from the high latitude origins, such as Hokkaido, Tohoku, and Hokuriku, have less photoperiod sensitivity and are expected to have a smaller estimated value of β. In contrast, a larger β should be observed for the photoperiod sensitive genotypes, mostly from the south of Japan, such as Kinki, Chugoku, and Kyushu. The average values of the posterior mean of β were approximately 1.01–1.48 for high latitude origins and 4.76–5.66 for low latitude origins. For α, we can find fewer differences between the average value of the posterior mean among origins (all average values were approximately 0.8–1.2). This probably reflects that the heading date in rice is more sensitive to the variation in photoperiod rather than the variation in temperature under the usual conditions. For parameter *G*, the smallest average posterior mean could be observed for the genotype of Northeast origins, Hokkaido (47.6 days) and a larger average value for the genotypes from Tohoku and Hokuriku (61.4 and 59.6 days), which are south of Hokkaido and in the north of Japan. The genotypes from the other origins had a similar average posterior mean of around 52.9–55.4 days.

**TABLE 3:** Average of the posterior statistics among 112 cultivars/lines from seven different origins.

**Parameters in DVR**	**Posterior**	**Cultivar origins**						
		**Hokkaido (9)**	**Tohoku (26)**	**Kanto and Tokai (24)**	**Hokuriku (14)**	**Kinki and Chugoku (9)**	**Kyushu (11)**	**Landrace and others (19)**
α	mean	0.792	1.062	1.141	1.224	0.944	0.891	0.976
	median	0.789	1.063	1.149	1.225	0.953	0.911	0.977
	mode	0.781	1.071	1.031	1.219	0.735	0.592	0.853
β	mean	1.478	1.015	3.994	1.336	4.764	5.652	4.499
	median	1.419	0.917	3.853	1.282	4.537	5.144	4.361
	mode	1.019	0.427	5.948	1.116	6.632	9.810	5.315
*G*	mean	47.559	61.432	55.236	59.62	55.435	54.788	52.916
	median	47.653	61.619	55.693	59.727	56.195	56.497	53.18
	mode	47.430	62.403	45.357	55.98	50.004	42.844	49.600

*The parameters α, β, and G in the DVR model represent the temperature sensitivity coefficient, the photoperiod coefficient, and the earliness of flowering under the optimal condition, respectively.*

### Comparison of Prediction Ability Between the Methods

The comparison between the prediction ability of the CGM, the machine learning approach, and the integrated approach for the heading date of rice is summarized in Table 4. We first evaluated the prediction of the heading date of a tested cultivar/line in a tested location through a fivefold CV process. The machine learning approach using XGB had a smaller RMSE (4.372 and 2.653 for the model using the environmental data with 14 heading date-related markers and 1,594 markers data, respectively, as input) than the CGM (5.711 for the DVR with the parameters estimated by the Bayesian approach with the DREAM algorithm). This shows that the use of environmental data and genetic data combined with the powerful machine learning method can better predict the heading date of the tested cultivar/line in a tested environment than the CGM alone. We then evaluated the prediction of the heading date of an untested genotype in a tested location using the LOGO CV process. As described in “Materials and Methods” section, the CGM requires parameters that are genotypic specific and is unable to make such predictions. The machine learning method XGB had a better predictive ability (RMSE = 5.02 and 4.468 with 14 heading date-related markers and 1,594 markers, respectively) in LOGO CV than the integrated approach (RMSE = 6.47 and 9.05 with 14 heading date-related markers and 1,594 markers, respectively). This shows that the single machine learning method could be a better predictor when the environmental data is included and the genotypic data are removed from the training data. The integrated approach achieved the prediction of the heading date of an untested genotype via the estimation of the CGM parameters and then via the fitting of the CGM with the estimates. Both the bias in predicting the CGM parameters and the adoption of relatively simple functions in the CGM compared to the more complex and flexible machine learning methods could be responsible for the relatively poor predictability in the integrated approach. The integrated approach shows its superiority in predicting the untested genotype in the untested location in the LOGLO CV process. The integrated approach adopted the Bayesian approach for the estimation of CGM parameters and trained an XGB model for predicting the parameters from genotype markers in step 1. Then, the heading date was predicted with the CGM of the predicted parameters in step 2. The procedure of this prediction, abbreviated as CGM-XGB, had the best predictive ability (RMSE = 7.69 when using 14 heading-related markers in machine learning) compared to a simple XGB model (RMSE = 9.361 and 8.537 for the model using the environmental data with 14 heading-related markers and 1,594 markers data, respectively, as input). In LOGLO CV, the information of the tested genotype and the tested location are removed from the training data, leading the predictor trained by the machine learning method to be more specific to the involved regions only. In contrast, the CGM quantifies the response of a plant to environmental factors using non-linear mechanical equations, which are more simplified but could be more robust in the prediction under a more uncertain condition.

**TABLE 4:** Root mean square errors (RMSE) of the three prediction methods used.

	**Crop growth model**	**Machine learning**		**Integrated approach**	
	**DVR^a^**	**XGB^b^**		**CGM-XGB^c^**	
		**14H**	**1,594**	**14H**	**1,594**
Fivefold^d^	5.711	4.372	2.653		
LOGO^e^		5.025	4.468	6.471	9.050
LOGLO^f^		9.361	8.573	7.690	9.793

*^*a*^DVR: DVR model with Bayesian DREAM Markov chain Monte-Carlo algorithm. ^*b*^XGB: gradient boosting method. ^*c*^CGM-XGB: the integrated approach combining DVR with XGB. ^*d*^Fivefold: fivefold cross-validation. ^*e*^LOGO: leave-one-genotype-out cross-validation. ^*f*^LOGLO: leave-one combination-of-genotype-and-location-out cross-validation. 14H represents 14 heading-related markers. 1,594 represents 1,594 markers, including the 14 heading-related markers.*

Table 5 shows the results of the integrated approaches that were implemented with the combinations of three machine learning methods (RF, XGB, and ELM), and two sets of genotype marker data (14 heading-related markers and 1,594 markers). First, we found that the model with 14 heading date-related markers had better prediction ability than the model with 1,594 markers, which also included the 14 heading-related markers. The lower prediction ability in the model with a larger number of markers could be attributed to the inclusion of markers irrelevant to phenological growth and the lack of training data for the target genotype. αβ*G* Second, the adoption of a different machine learning method could affect the prediction ability. The rank of the ability in the model was XGB > ELM > RF with 14 heading date-related markers, and RF > XGB > ELM with the 1,564 markers. It reveals that XGB and ELM could be a better predictor of CGM parameters when less noise is present in the input data (14 heading date-related markers), whereas RF is relatively robust to the input data with noise. ELM could be greatly affected by the noise in the input data and even provided a highly deviated estimation of the parameters. Such problems could be found in the especially large RMSE of ELM in the model with 1,594 markers. Among all combinations of methods in the integrated approaches, XGB in step 1b in the integrated approach had the best prediction ability in both the LOGO and LOGLO CV processes.

**TABLE 5:** Root mean square errors (RMSE) of the integrated approaches involving three different machine learning methods.

	**Methods in step 1a of the integrated approaches^a^**		
	
**Methods in step 1b of the integrated approaches^b^**		**Bayesian with normal dist. prior**	
		
	**Marker**	**LOGO^c^**	**LOGLO^d^**
ELM	14H	6.566	7.731
XGB	14H	6.574	7.776
RF	14H	6.817	8.038
ELM	1,594	18.627	19.087
XGB	1,594	9.552	10.658
RF	1,594	7.716	8.528

*^*a*^Step 1 in the integrated approaches was to estimate the crop growth model parameters using the rice heading date data and the environmental data. ^*b*^Step 2 in the integrated approaches was to train a machine learning model to predict the CGM parameters of an unknown genotype. ^*c*^LOGO: leave-one-genotype-out cross-validation. ^*d*^LOGLO: leave-one-combination-of-genotype-and-location-out cross-validation. ELM, extreme learning machine; XGB, eXtreme gradient boosting; RF, random forest.*

### Predicting DTH Distribution in F_2_ Segregation Populations

**FIGURE 2:**
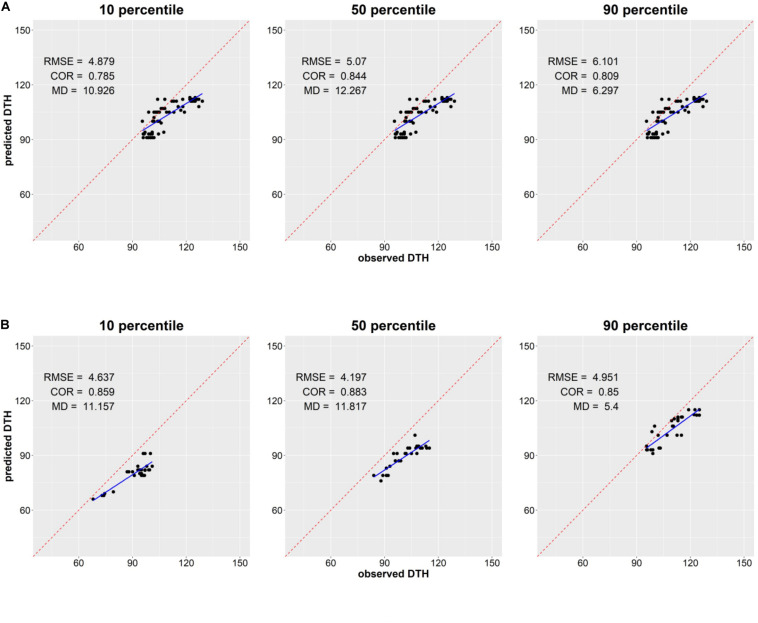
Scatterplot of the 10, 50, and 90th percentiles of observed and predicted distribution of days to heading (DTH) of segregation populations in 2008 **(A)** and in 2009 **(B)**. The observed DTH was obtained from the 96 plants of each population, and the predicted DTH was generated from the proposed integrated approach CGM-XGB. RMSE: root mean square error; COR: correlation coefficient; MD: mean absolute difference.

We examined the ability of the proposed integrated model CGM-XGB in predicting the distribution of DTH in 103 F_2_ segregation populations. Figure 2 shows the scatterplot of the 10, 50, and 90th percentiles of the observed distributions and predicted distributions. The predicted RMSE, correlation coefficient, and absolute mean difference are also shown in Figure 2. The percentiles of the predicted DTH distribution tended to be underestimated in comparison to the percentiles of the observed DTH distribution for most populations. The correlation coefficients were mostly over 0.8, and showed that the integrated approach could be useful in predicting the rank of the percentiles of distribution in DTH between different F_2_ segregation populations. A slightly better prediction was found in the 30 populations tested in 2009 than in the 73 populations tested in 2008, although the reason for this is unclear. Histograms of the observed and predicted distributions in DTH for each segregation population are shown in Supplementary Figures S1–S3.

## Discussion

In this study, we proposed a potential integrated approach that combines machine learning methods and a CGM to improve the modeling of physiological growth of rice plants. We emphasize the importance of the training data for the successful building of the model. Phenotypic and environmental data consisting of a wide range of genotypes grown in multiple environments is a prerequisite for the proposed approach. In this study, 112 cultivars/lines were selected from among those adapted to different ecological regions in Japan (Yamasaki and Ideta, 2013) and had been evaluated at these locations for more than 10 years. Such comprehensive data allows us to estimate the phenological parameters in a CGM with less estimation bias and mitigates the bias induced by the location effect and makes it possible to associate the marker effect with the model parameters. In addition, the real data of the F_2_ segregating populations presents opportunities to validate the predictability of the model for predicting the potential of a cross to develop a new cultivar/line for a new environment. In previous studies, this validation was mostly conducted using a simulation study or cross-validation that might not reflect the true performance of the proposed model. The power of machine learning methods is in addition to the quality of the training data. Because more information can be collected from high-throughput phenotyping, genotyping, environmental sensing, and omics analyses, more attention can be paid to the data rather than only the methodologies.

Estimating the parameters of the CGM appropriately is essential for the prediction accuracy of a model and for further inference that utilizes the predicted model parameters. Despite prior knowledge, the estimation method can influence the results; therefore, the best strategy to conduct such estimation remains open for discussion. For parameter estimation, we implemented both frequentist and Bayesian approaches and showed no obvious difference in the prediction accuracy of the CMG (results not shown here). This might mainly result from the substantial and complete heading data collected in this study, which provides sufficient information for parameter estimation.

The parameters α, β, and G in the DVR model represent the temperature sensitivity coefficient, the photoperiod coefficient, and the earliness of flowering under the optimal condition, respectively. In this study, we obtained not only the point estimated value but also the approximated posterior distribution of these three parameters for 112 Japanese rice cultivars. This information allowed us to first examine the phenological characteristics of the most representative cultivars quantitatively and use them in building the integrated model. The characteristics of most Japanese rice cultivars, including the tendency of photoperiod sensitivity, can be found in a database^1^. We compared the tendency of photoperiod sensitivity of the tested cultivars/lines to the posterior mean of β, and the results were mostly matched (results not shown here). In addition, parameter estimation using the Bayesian method also matched our knowledge regarding the character of a genotype and might better quantify the indirect features of the phenological growth of rice.

As shown in Table 3, the β of cultivars originating in high latitude regions was close to 1, indicating the strong tendency of photoperiod insensitivity and vice versa. The results are consistent with those of a previous study (Okumoto et al., 1996) and rice photoperiod sensitivity is generally diverse (Hori et al., 2016). All five heading date-related genes in this study are associated with the rice photoperiodic pathway (Yano et al., 2000; Takahashi et al., 2001; Xue et al., 2008; Matsubara et al., 2012; Hori et al., 2013). Although temperature is also an essential factor in predicting rice growth, the variation in α was small, suggesting that the diversity of the thermal reaction among the 112 cultivars may be small. In combination with the estimation of α and G, it presented the possible coordination between thermal reaction, photoperiod sensitivity, and the earliness of flower initiation that helps the corresponding cultivar to adapt to the target environment.

Compared to the results achieved by machine learning methods in other fields in agriculture, such as crop management and water management (Liakos et al., 2018), examples of successful applications in crop breeding and genetics are still relatively rare. The fundamental reason is not only the complexity of the genotype × environment × management interaction, but also the unfamiliarity of the method, the lack of adequate data, and the few experts who are familiar with both fields. We compared the use of a CGM, machine learning models, and integrated approaches in predicting rice heading. The results showed that the machine learning model with the genotypic marker was more accurate than the CGM in predicting the heading of a tested cultivar/line in a tested location. We also compared the predictability of three machine learning methods: RF (a popular ensemble learning method), ELM (a feed-forward neural network), and XGB (a modified gradient boosting method), and showed the advantages of applying the newly developed algorithm. It is not surprising that the machine learning methods were capable of better capturing the complex and non-linear association between complicated traits and genetic and environmental variables. However, at the same time, a machine learning method could yield worse predictions than a mechanistic CGM if the training data is limited or full of noise. We also showed that the machine learning models were less applicable for predicting the extrapolation problem, such as the prediction of the heading of untested genotypes in an untested location that can be predicted better by the proposed integrated approach. However, both CGM and the machine learning model could be useful for cultivation management, such as supporting the decision on the suitable sowing timing for an optimal heading date.

The three machine learning methods (RF, XGB, and ELM) compared in this study have proven their superiority in many machine learning challenges, and the implemented packages are already available to run on many platforms. Although XGB and ELM had slightly better predictability than RF in our results, there is no guarantee that one method could outperform others in a different scenario. The experimental design, training data, and setting of hyperparameters sometimes play an important role in practical applications. In addition, factors such as (1) suitability to a given setting, (2) computational cost, (3) software availability, and (4) usability, may be considered when selecting the best method (Sagi and Rokach, 2018). In addition, the lack of interpretability in most machine learning methods could be an issue when we apply them to biological problems. For example, the machine learning model in our integrated approach could not provide an intuitive understanding of the underlying gene regulation of rice heading. The development of interpretable machine learning methods might be helpful in the future when both predictability and interpretability are needed.

Using 112 cultivars, the integrated model CGM-XGB simulated and predicted the distributions of DTH in 103 F_2_ segregation populations. The predicted distributions of DTH were generally similar to those observed in the real data. Based on the prediction of DTH in a segregating population in an environment and management system before producing crosses, breeders can consider the optimum cross combinations to develop a novel cultivar. In addition, a recent serious event, high temperature during the rice ripening period resulted in deterioration of the grain quality in Japan (Morita, 2009). The models explored in this study can propose the ideal heading date and sowing timing in a cultivar to avoid such damage.

## Conclusion

The capability of the proposed integrated approach in predicting the heading of a new genotype in a new environment was demonstrated, and this could prove useful in suggesting the locally adapted ideotype for rice phenology. We also revealed that the machine learning model could outperform the crop growth model (CGM) (phenological model without genotypic data) in predicting the heading of a tested cultivar/line in a tested environment and could be replaced with a phenological model when higher accuracy is preferred. However, the machine learning model is highly dependent on the given data and is usually less capable of extrapolating, as demonstrated by the Leave-one-genotype-out cross-validation (LOGLO CV) results. It is also difficult to dissect the machine learning model and reveal the explanatory mechanisms underneath the model, as can be done with the CGM. The CGM models the key physiological processes of crop growth, and the inclusion of CGM into the modeling platform can reduce the uncertainty when simulating crop growth. This study confirmed that the integrated approach improved the prediction of the complex trait for a new genotype in a new location and may benefit crop selection.

## Data Availability Statement

The data analyzed in this study is subject to the following licenses/restrictions: The dataset analyzed in this study is not publicly available due to parallel studies but may be available from the corresponding author upon reasonable request. Requests to access these datasets should be directed to Hiroyoshi Iwata, hiroiwata@g.ecc.u-tokyo.ac.jp.

## Author Contributions

T-SC developed the methodology, analyzed the data, wrote the manuscript, and developed the software. MY designed and conducted the experiments and provided the rice heading data and marker data. HK-K prepared and provided marker data. TA conducted the pioneer study and was involved in methodology development. HI was involved in the conceptualization, methodology development, and supervision of the study. All authors contributed to the article and approved the submitted version.

## Conflict of Interest

The authors declare that the research was conducted in the absence of any commercial or financial relationships that could be construed as a potential conflict of interest.

## Footnotes

1 https://ineweb.narcc.affrc.go.jp/
