# Supplementary material for: Predicting Rice Heading Date Using an Integrated Approach Combining a Machine Learning Method and a Crop Growth Model
Source: Front Genet. 2020 Dec 18;11:599510. doi: 10.3389/fgene.2020.599510 (PMC7775545; doi:10.3389/fgene.2020.599510)
Supplement: Supplementary file 1 [file Data_Sheet_1.docx]

Supplementary Material

# Appendix

**Estimation of crop model parameters using the differential evolution adaptive metropolis (DREAM) algorithm**

The DREAM algorithm (Vrugt et al., 2009) was derived from the differential evolution Markov chain (DE-MC) algorithm, but includes subspace sampling and outlier chain correction to accelerate the convergence to the target distribution. It has shown its superiority to existing Markov chain Monte-Carlo schemes and can efficiently handle the problems involving non-linearity, high dimensionality, and multimodality. In this study, we implemented the DREAM algorithm to estimate the Developmental rate (DVR) model parameters with the following steps.

1. Determine the hyperparameters in DREAM: the number of chains *N* = 10, the number of generations *T*= 50,000, the number of chain pairs proposal $\delta=3$, and the number of crossover values $n_{cr}$ = 3.
2. Determine the prior and likelihood functions. The non-informative prior was adopted in this study. The upper limit and lower boundaries of the DVR model parameters are listed in Table 2.

We assumed that the errors (residuals) are uncorrelated and followed the Gaussian distribution; the likelihood function is

$$L\left( Y | \theta\right)=\prod_{i=1}^{n} \frac{1}{\sqrt{2\pi\sigma_{i}^{2}}}exp\left\{ \frac{\left[ \hat{y_{i}}\left( \theta,X \right)-y_{i} \right]^{2}}{2\sigma_{i}^{2}} \right\}$$

where $\sigma_{i}^{2}$ is the error variance of the measurement $i$.

Following conventional practice, we used the logarithm of the likelihood function:

$$\ln\left( L\left( Y | \theta\right) \right)=-\left( \frac{n}{2} \right)\ln\left( 2\pi\right)-\sum_{i=1}^{n} \ln\left( \sigma_{i} \right)-(1/2)\sum_{i=1}^{n} \left( \frac{\hat{y_{i}}\left( \theta,X \right)-y_{i}}{\sigma_{i}} \right)^{2}$$

1. Draw an initial population X using the prior distribution. X is a $d\times N$ matrix with columns {$x^{i}, i=1,\ldots,N$}. $d$ is the number of parameters.
2. Compute the prior density $g(x^{i})$ for $i=1,\ldots,N$. **(start of chain evolution)**
3. Generate the candidate point $X_{p}^{i}$ in the chain $i$ using the DE-MC algorithm, but with subset sampling and outlier correction.

If $A$ is a subset of $d^{'}$ dimensions of the original parameter space, $R^{d^{'}}\subseteq R^{d}$, then a jump, $dX^{i}$, in the $i$th chain at iteration $t$ is calculated from the collection of chains X using differential evolution:

$$dX_{A}^{i}=\zeta_{d^{'}}+\left( 1_{d^{'}}+\lambda_{d^{'}} \right)\gamma\left( \delta,d^{'} \right)\left( \sum_{j=1}^{\delta} X_{A}^{a_{j}}-X_{A}^{b_{j}} \right)$$

$$dX_{\neq A}^{i}=0$$

where $\gamma\left( \delta,d^{'} \right)=2.38/\sqrt{2\delta d^{'}}$ is the jump rate, denoting the number of chain pairs used to generate the jump, and $a$ and $b$ are vectors comprising $\delta$ integers drawn without replacement from $\left\{ 1,\ldots,i-1,i+1,\ldots,N \right\}.$ The values of $\lambda$ and $\zeta$ are sampled independently from the multivariate uniform distribution $u_{d^{'}}\left( -c,c \right)$ and multivariate normal distribution $N(0,c_{*})$, respectively. $c$ and $c_{*}$ are set to 0.1 and ${10}^{-12}$, respectively.

Then, the candidate point of chain $i$ at iteration t becomes

$$X_{p}^{i}=X^{i}+dX^{i}$$

1. Replace each element $\left( j=1,\ldots,d \right)$ of the proposal ${X_{p}^{i}}_{j}$ with ${X^{i}}_{j}$ using a binomial scheme with probability $1-CR$, where $CR$ is the crossover probability. With $CR=1$, all dimensions are updated jointly and $d’=d$.

$${X_{p}^{i}}_{j}=\left\{ \begin{aligned} {X^{i}}_{j}\text{ if }U\leq1-CR\text{, }d^{'}=d^{'}-1 \\ {X_{p}^{i}}_{j}\text{ otherwise } \end{aligned} j=1,\ldots,d \right.$$

where $U\in\left[ 0,1 \right]$ is a drawn from a uniform distribution.

1. Calculate the metropolis acceptance probability:

$$P_{acc}\left( X^{i}\to X_{p}^{i} \right)=min\left( 1,\frac{p\left( X_{p}^{i} \right)}{p\left( X^{i} \right)} \right)$$

2. If $P_{acc}\left( X^{i}\to X_{p}^{i} \right)\geq U$, the candidate point is accepted and the $i$th chain moves to the new position, $x_{t}^{i}=X_{P}^{i}$, otherwise $x_{t}^{i}=x_{t-1}^{i}$ **(end of chain evolution)**
3. Remove outlier chains using the inter-quartile-range statistic. This is done during burning. (optional)

The Julia program written for the above algorithm can be found at the following link [https://github.com/billy0205/DREAM_MCMC_CGM.git]

**Calculation of theoretical day length using the CBM model**

There are three steps in the CBM model. The first step is predicting the revolution angle (θ) from the day of the year ($J$).

$$\theta=0.2163108+2\tan^{-1} [0.9671396\tan[0.00860\times(J-186)]]$$

The second step is to predict the sun’s declination angle ($\varphi$)

$$\varphi=\sin^{-1} [0.39795\cos\theta]$$

The third step is predicting day length (D) from latitude (L), longitude, and the sun’s declination angle ($\varphi$).

$$D=24-\frac{24}{\pi}\cos^{-1} [\frac{\sin\frac{p\pi}{180}+\sin\frac{L\pi}{180}\sin\varphi}{\cos\frac{L\pi}{180}\cos\varphi}]$$

where $p$ is defined as 6 that reflects the definition of day length including civil twilight.

Supplementary Figure **
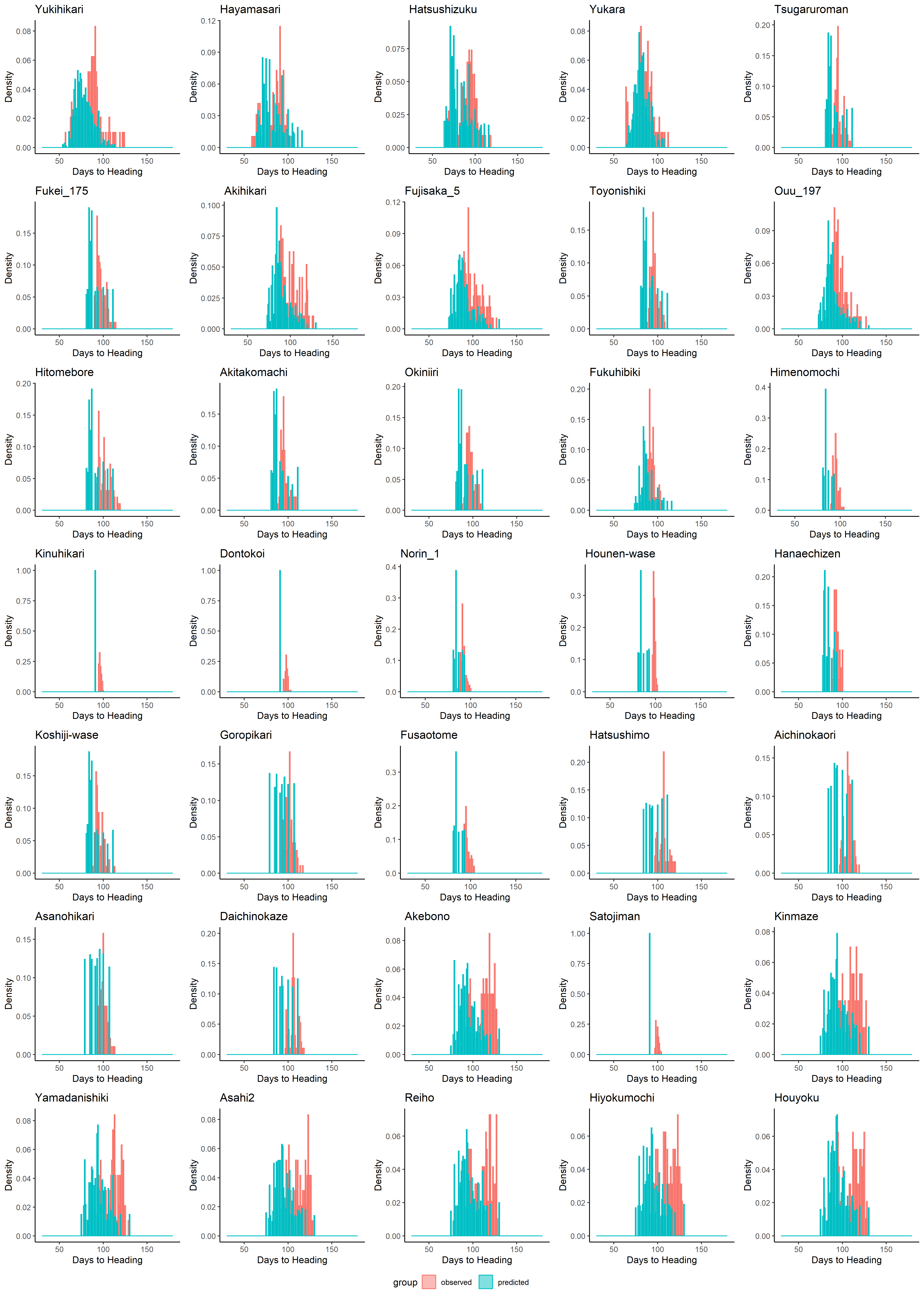
**

**Supplementary Figure 1.** Histograms of the observed and predicted distribution in DTH for the F_2_ segregation populations in 2008 (35 out of 73 populations in 2008).

The F_2_ segregation population was created by crossing ‘Koshihikari’ and the selected cultivars. The name of the selected cultivars is shown in the label in each histogram. The red distribution represents the observed DTH of 96 plants. The blue distribution represents the predicted DTH of 1000 simulated genotypes obtained from the proposed integrated approach.


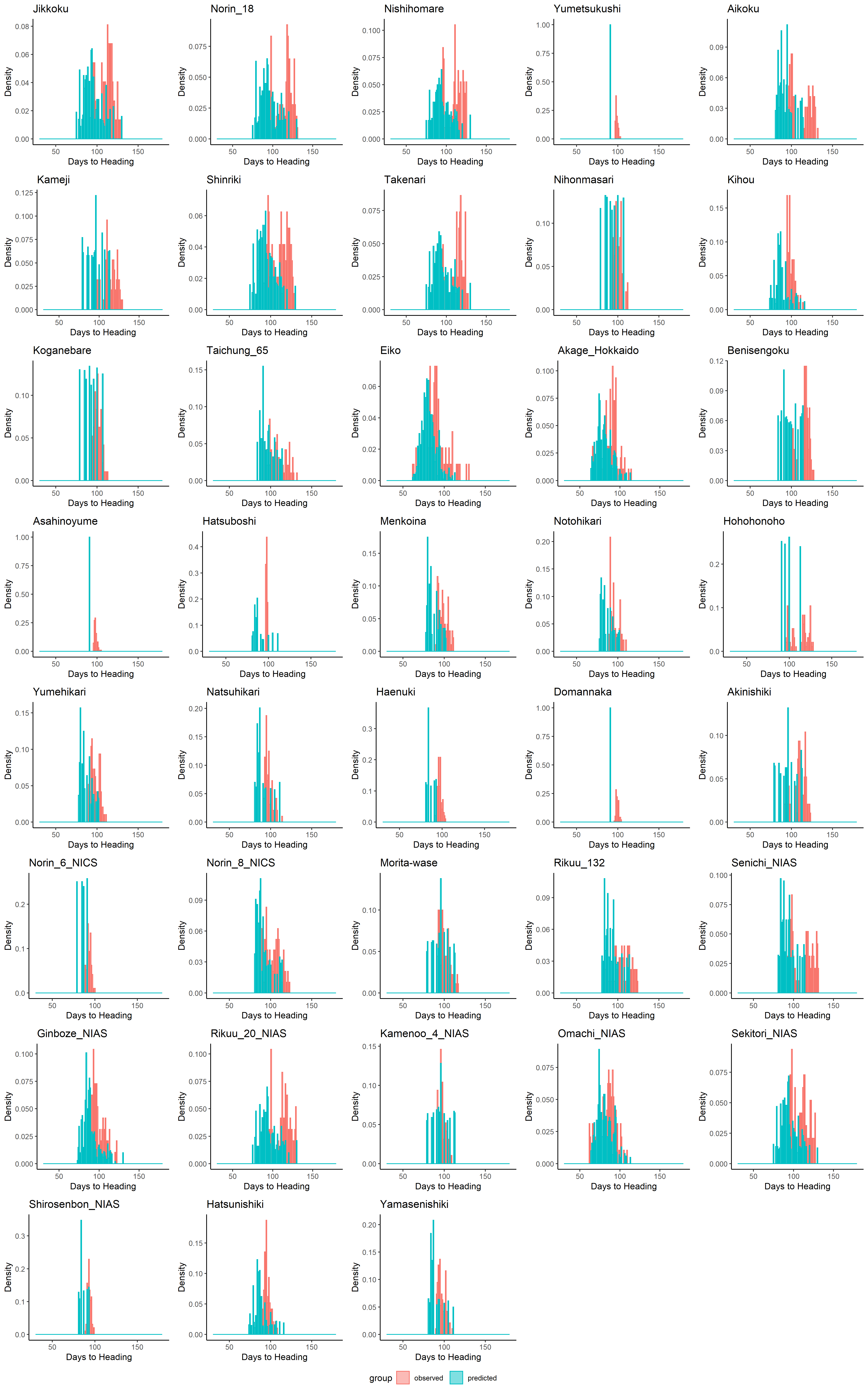


**Supplementary Figure 2.** Histograms of the observed and predicted distribution in DTH for the F_2_ segregation populations in 2008 (the remaining 33 out of 73 populations in 2008).


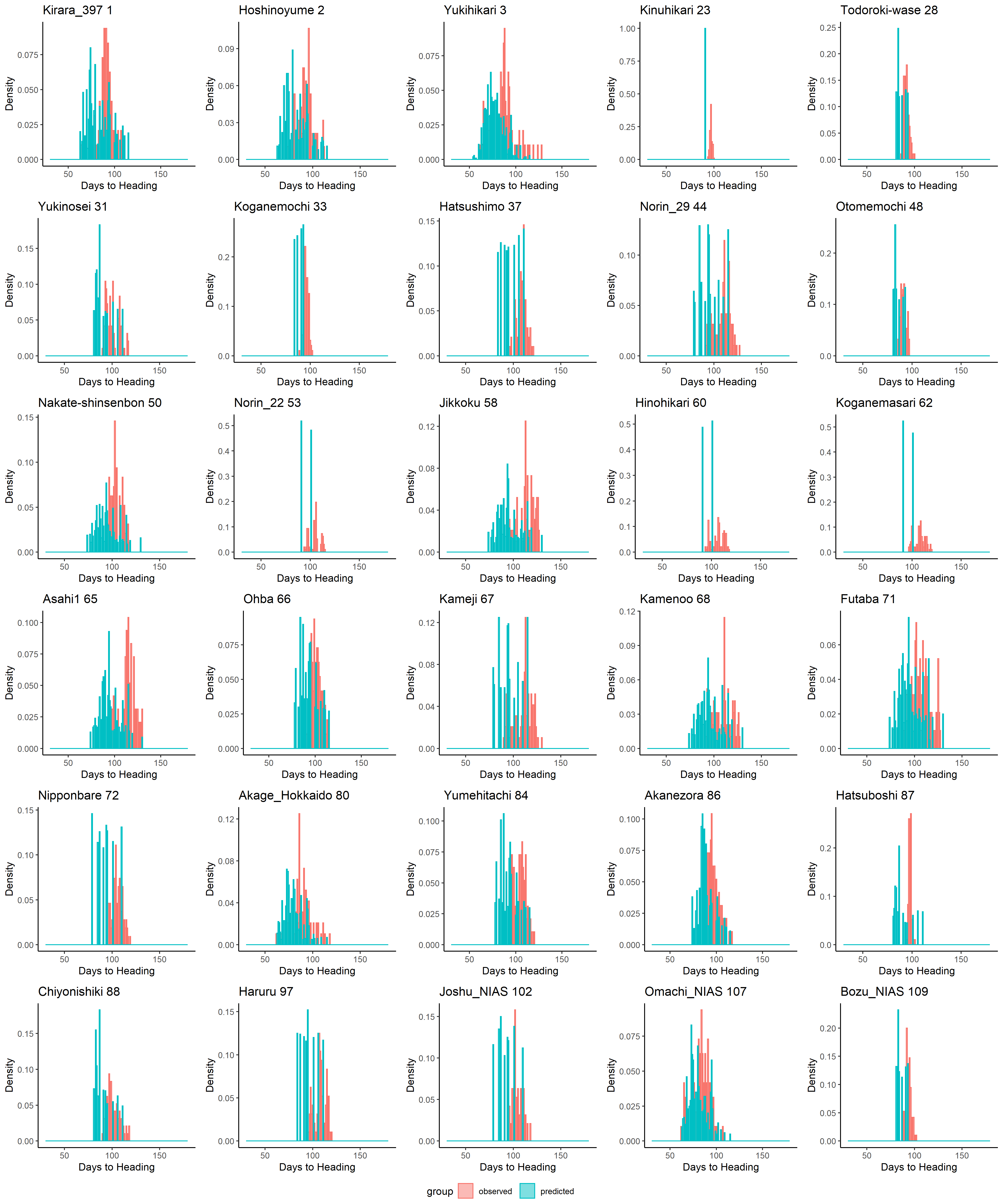


**Supplementary Figure 3.** Histograms of the observed and predicted distribution in DTH for 30 F_2_ segregation populations in 2009.
